# Imoxin attenuates LPS‐induced inflammation and MuRF1 expression in mouse skeletal muscle

**DOI:** 10.14814/phy2.13941

**Published:** 2018-12-09

**Authors:** Rudy J. Valentine, Matthew A. Jefferson, Marian L. Kohut, Hyeyoon Eo

**Affiliations:** ^1^ Department of Kinesiology Iowa State University Ames Iowa; ^2^ Interdepartmental Graduate Program in Nutritional Sciences Iowa State University Ames Iowa; ^3^ Immunobiology Interdepartmental Graduate Program Iowa State University Ames Iowa; ^4^ Interdepartmental Neuroscience Graduate Program Iowa State University Ames Iowa

**Keywords:** Atrophy, C16, cytokine, endotoxin, PKR, skeletal muscle

## Abstract

The double‐stranded RNA‐dependent protein kinase (PKR) contributes to inflammatory cytokine expression and disease pathogenesis in many conditions. Limited data are available on the efficacy of the PKR inhibitor imoxin to prevent lipopolysaccharide (LPS)‐induced inflammation in skeletal muscle in vivo. The aim of this study was to evaluate the effect of imoxin, a PKR inhibitor, on inflammatory and atrophy signaling in skeletal muscle in response to an acute inflammatory insult with LPS. Six‐week old C57BL/6J mice received vehicle (saline) or 0.5 mg/kg imoxin 24 and 2 h prior to induction of inflammation via 1 mg/kg LPS. Gastrocnemius muscles were collected 24 h post‐LPS and mRNA and protein expression were assessed. LPS lead to a loss of body weight, which was similar in Imoxin+LPS. There were no differences in muscle weight among groups. LPS increased gastrocnemius mRNA expression of TNF‐*α* and IL‐1*β*, and protein levels of NLRP3, all of which were attenuated by imoxin. Similarly, IL‐6 mRNA and IL‐1*β* protein were suppressed in Imoxin+LPS compared to LPS alone. LPS increased mRNA of the atrogenes, MuRF1 and MAFbx, and imoxin attenuated the LPS‐induced increase in MuRF1 mRNA, and lowered MuRF1 protein. Imoxin+LPS increased p‐Akt compared to saline or LPS, whereas p‐mTOR was unaltered. FoxO1 was upregulated and p‐FoxO1/FoxO1 reduced by LPS, both of which were prevented by imoxin. Both LPS and Imoxin+LPS had diminished p‐FoxO3/FoxO3 compared to control. These results demonstrate the potential anti‐inflammatory and anti‐atrophy effects of imoxin on skeletal muscle in vivo.

## Introduction

Skeletal muscle inflammation occurs in a variety of conditions in which muscle loss is a complication (Costamagna et al. 2015; Powers et al. 2016). A feature common in many muscle wasting conditions is an elevation in circulating endotoxin. High concentrations of endotoxin, or lipopolysaccharide (LPS), have been observed in obesity, insulin resistance, type 2 diabetes (Cani et al. 2008; Al‐Attas et al. 2009; Ghanim et al. 2009; Miller et al. 2009), NAFLD (Harte et al. 2010), cancer (Han et al. 2007), and chronic heart failure (Niebauer et al. 1999), likely contributing to the muscle wasting observed in these conditions. The administration of lipopolysaccharide (LPS) is an established model that reproducibly induces skeletal muscle inflammation (Frost et al. 2002; Lang et al. 2003; Frost and Lang 2005; Huey et al. 2008a) and muscle atrophy gene expression (Dehoux et al. 2003). However, a full understanding of the key players and events that coordinate the complex catabolic signaling response to LPS is still elusive.

The double‐stranded RNA‐dependent protein kinase (PKR) contributes to inflammatory cytokine production (Kang and Tang 2012) and metabolic dysregulation (Nakamura et al. 2010). Indicators of PKR activity are elevated in skeletal muscle in models of atrophy, including cancer (Eley et al. 2008d) and diabetic conditions (Russell et al. 2009; Carvalho et al. 2013). In in vitro, stimulation with LPS, along with other atrophic agents, increases PKR activity, resulting in activation of the ubiquitin proteasome system (UPS) (Eley and Tisdale 2007; Eley et al. 2008a,b,c, 2010; Supinski and Callahan 2011). Additionally, PKR suppresses protein synthesis by phosphorylating, and inhibiting, eIF2*α*, a translational initiation factor (Dey et al. 2005). Genetic inactivation of PKR prevents the phosphorylation of eIF2a, resulting in restoration of protein synthesis and suppression of protein degradation (Eley and Tisdale 2007; Eley et al. 2008a,b; Supinski and Callahan 2011). Importantly, pharmacological inhibition of PKR with imoxin, also referred to as compound 16 (C16), appears to recapitulate the genetic effects, and has been shown to have anti‐inflammatory effects in multiple tissues (Supinski and Callahan 2011; Tronel et al. 2014; Nakamura et al. 2015; Li et al. 2017). However, in some instances, such as in response to viral infection, PKR may be anti‐inflammatory (Kapil et al. 2014). The role of PKR in inflammation appears to be dependent on both context and cell type (Yoshida et al. 2012; Watanabe et al. 2018).

Whether PKR is involved in skeletal muscle inflammatory and atrophy responses to LPS, in vivo, is not established. Additionally, skeletal muscle signaling responses that may be impacted by PKR in response to LPS have not been fully characterized. In this context, the primary objective of the current study was to evaluate the protective effect of imoxin, in vivo, against LPS‐induced skeletal muscle inflammation. We hypothesized that the PKR inhibitor imoxin would alleviate the effects of LPS on skeletal muscle inflammation and atrophy signaling.

## Materials and Methods

### Experimental design

Male C57BL/6J mice were purchased from Jackson Laboratories (Bar Harbor, ME) at 5 weeks of age. Mice were group housed on a 12:12‐h light‐dark cycle in a temperature‐controlled (19–21°C) room at 4–5 mice per cage with free access to water and standard chow. Mice were acclimated for 1 week prior to experiments, then randomly assigned to saline+saline (control), saline+LPS (LPS), or Imoxin+LPS groups (*n* = 6–7 per group). Mice received two subcutaneous (s.c.) injections of sterile saline or 0.5 mg/kg imoxin (Calbiochem, San Diego, CA) given 24 and 2 h prior to an LPS challenge. Imoxin was reconstituted at 20 mmol/L in 100% EtOH (5.37 mg/mL), and further diluted in saline to 0.1 mg/mL, and injected s.c. (~0.1 mL/mouse). The dose of imoxin was selected based on previous work demonstrating anti‐inflammatory and metabolic benefits with similar doses (Tronel et al. 2014; Li et al. 2017), without noticeable ill effects, even with prolonged use (Nakamura et al. 2014). Sterile saline or LPS (serotype O111:B4, Sigma‐Aldrich, St. Louis, MO) was injected intraperitoneally (i.p.) at a dose of 1 mg/kg in a 0.1 mg/mL saline solution, ~0.2 mL/mouse. The dose of LPS was selected to induce skeletal muscle inflammation, without inducing sepsis, overt muscle atrophy, or risk of mortality (Huey et al. 2008b; Li et al. 2015). Twenty‐four hours after LPS administration, mice were euthanized by carbon dioxide inhalation, and gastrocnemius muscles were quickly removed, trimmed of excess fat and connective tissue, wet weighed, and snap‐frozen in liquid nitrogen. Each gastrocnemius was processed for both protein and mRNA, as described below. Protocols for animal use were reviewed and approved by the Institutional Animal Care and Use Committee of Iowa State University and were in accordance with the American Physiological Society Animal Care Guidelines.

### Chemicals and materials

Antibodies for NLRP3 (#13158), p‐Akt (Ser 473) (#9721), Akt (#9272), p‐eIF2*α* (Ser 51) (#3398), p‐FoxO1/p‐FoxO3 (Thr 24/Thr 32) (#9464), p‐mTOR (Ser 2448) (#2971), mTOR (#2983), eIF2*α* (#5324), p‐NF‐*κ*B (Ser 536) (#3033), I*κ*B*α* (#4814), and ubiquitin (#3933) were obtained from Cell Signaling (Danvers, MA) and p‐PKR (Thr 451) (#44‐668) was from Invitrogen (Carlsbad, CA). Mouse monoclonal IL‐1*β* (sc‐1251), TNF‐*α* (sc‐52746), PKR (sc‐6282), MuRF1 (sc‐398608), MAFbx (sc‐166806), FoxO1 (sc‐374427), FoxO3 (sc‐48348), and NF‐*κ*B (sc‐8008) were from Santa Cruz Biotechnology (Santa Cruz, CA). All other chemicals were purchased from Thermo Fisher Scientific (Waltham, MA), unless otherwise indicated.

### mRNA analysis

Gastroc samples (~50 mg) were homogenized in 1 mL TRIzol. Total RNA was extracted and concentration was quantified spectrophotometrically using the NanoDrop system (NanoDrop Technologies, Wilmington, DE). Total RNA (1 *μ*g) was reverse transcribed to cDNA using a Verso cDNA Synthesis Kit (Thermo Scientific, Grand Island, NY) according to manufacturer instructions. Quantitative real‐time PCR was performed with 20 ng/reaction using iTaq Universal SYBR Green Supermix (Bio‐Rad, Hercules, CA), and run on a MyiQ PCR system (Bio‐Rad, Hercules, CA). Each gene was normalized against the housekeeping gene GAPDH, using the ΔΔC_T_ method, and mRNA expression for each gene is presented as mean fold‐change relative to the saline (control) mice. The primer sequences used for each gene are as follows: GAPDH forward: CCAGCTACTCGCGGCTTTA, reverse: GAGGGCTGCAGTCCGTATTT; TNF‐*α*: forward: AGCCGATGGGTTGTACCTTG, reverse: ATAGCAAATCGGCTGACGGT; IL‐6: forward: TCCAGTTGCCTTCTTGGGAC, reverse: AGTCTCCTCTCCGGACTTGT; IL‐1*β*: forward: TGCCACCTTTTGACAGTGATG, reverse: TGATGTGCTGCTGCGAGATT; NLRP3: forward: GACACGAGTCCTGGTGACTTT, reverse: CAGACGTATGTCCTGAGCCAT; MuRF1: forward: TGTCTCACGTGTGAGGTGCCTA, reverse: CACCAGCATGGAGATGCAGTTAC; MAFbx: forward: ACATTCTGCCAGCTGCTGTTTC, reverse: TGAGTTGGATGCTGGGCCTAC.

### Protein analysis

Frozen gastrocnemius samples (40–50 mg) were homogenized in 10× volume of ice‐cold buffer containing 30 mmol/L NaHepes (pH 7.4), 5 mmol/L EGTA, 3 mmol/L EDTA, 32% glycerol, 20 mmol/L KCl, HALT protease inhibitor cocktail (Thermo Fisher, Waltham, MA) and phosphate inhibitor cocktail 3 (Sigma‐Aldrich, St. Louis, MO). Tissue homogenates were centrifuged at 12,000*g* for 15 min at 4°C, and supernatants were collected. Protein concentration was assessed by the bicinchoninic acid method (BCA; Pierce Biotechnology, Inc., Rockford, IL), with BSA for the standard curve.

### Western blot analysis

Protein homogenates (15–30 *μ*g) were run on a 4–15% gradient Stain‐Free Criterion TGX gel (Bio‐Rad, Hercules, CA), and transferred onto a PVDF (polyvinylidene difluoride) membrane (EMD Millipore, Burlington, MA). Gels were activated according to Bio‐Rad's Stain‐Free protocol, and membranes were imaged to ensure even transfer and quantify total protein, as recommended (Ghosh et al. 2014), then blocked in Tris‐buffered saline (pH 7.5) containing 0.05% Tween‐20 (TBST) and 5% non‐fat dry milk for 1 h at room temperature. Next, they were incubated overnight in primary antibodies at a 1:1000 dilution. They were then washed with TBST, incubated with a secondary antibody conjugated to horseradish peroxidase (Cell Signaling) at a 1:5000 dilution, and subjected to an enhanced chemiluminescence solution. Western blot images were captured with the ChemiDoc™ XRS Imaging System (Bio‐Rad, Hercules, CA), and densitometry was performed using Image Lab V6.0 (Bio‐Rad, Hercules, CA).

### Statistics

Statistical differences between groups were determined by one‐way analysis of variance (ANOVA) with a Newman–Keuls post‐hoc test (Graphpad Prism; V6.01; GraphPad Software, Inc., CA). Data are reported as mean ± SEM and considered significant at *P* < 0.05.

## Results

### Body and muscle weight

Initial body weights were less in the Imoxin+LPS mice compared to LPS mice (*P *<* *0.05), but did not differ across remaining groups (Table**** 1). As expected, mice lost body weight (−2.7 ± 0.5 g), following LPS injection, which was not affected by imoxin pretreatment (−2.3 ± 0.7 g). In accordance with previous reports, absolute and relative gastrocnemius and soleus muscle weights were not affected by this dose of LPS (Huey et al. 2008b). To rule out an effect of imoxin alone on body weight and muscle weight, a subset of mice received twice the dose of imoxin (1.0 mg/kg), and weights were not altered (Table**** 1).

**Table 1:** Body and muscle weight

	Control	Imoxin 1 mg/kg	LPS	Imoxin 0.5 mg/kg + LPS
Body weight (pretreatment)	23.2 ± 0.6	22.3 ± 0.7	24.1 ± 0.6	21.8 ± 0.4#
Body weight change (%)	0.5 ± 0.5	2.1 ± 0.2	‐11.3 ± 0.8* †	‐10.2 ± 0.7* †
Gastrocnemius weight (mg)	140.6 ± 5.9	129.0 ± 6.1	130.7 ± 6.5	124.3 ± 3.0
Relative gastrocnemius weight (mg/g BW)	6.0 ± 0.2	5.7 ± 0.2	6.1 ± 0.2	6.4 ± 0.1

Body weight and weight loss 24 h after 1 mg/kg LPS injection. Weight of gastrocnemius muscles are averages of left and right limbs. Values are mean ± SE, *n* = 5–7 per group. *P < 0.05 compared to control, ^#^ *P* < 0.05 compared to LPS, ^†^ *P* < 0.05 compared to Imoxin 1 mg/kg.

### Muscle inflammatory mRNA and protein expression

**Figure 1:**
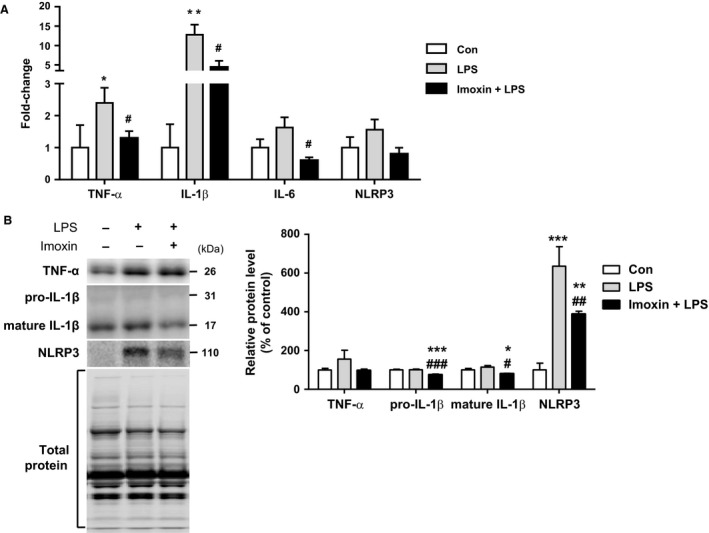
Inhibition of PKR with imoxin attenuates LPS‐induced skeletal muscle inflammation. Male C57BL/6J mice were injected s.c. with 0.5 mg/kg imoxin or saline 2 h and 24 h prior to 1 mg/kg LPS administered i.p. Gastrocnemius muscles were harvested 24 h following saline or LPS. Gene expression of inflammatory cytokines was determined by qPCR (A). Inflammatory proteins were assessed via western blot and representative blots and quantification are displayed (B). Data are expressed relative to saline control mice, and presented as mean ± SE (n = 6–7/group). *P < 0.05, **P < 0.01, and ***P < 0.001 compared with the Control group, #*P* < 0.05, ##*P* < 0.01, and ###*P* < 0.001 compared with the LPS group.

LPS increased the mRNA expression of TNF‐*α* and IL‐1*β*, by 2.4‐fold and 12.8‐fold, respectively, compared to saline‐treated mice. Pretreatment with imoxin attenuated the LPS‐induced increases in both, by 46% and 66%, respectively (*P* < 0.05; Fig. 1A). Although LPS did not significantly increase IL‐6 mRNA expression, levels were 63% lower in Imoxin+LPS compared to LPS alone (*P* < 0.05). There were no differences among groups on NLRP3 gene expression (ANOVA *P* = 0.26).

**Figure 2:**
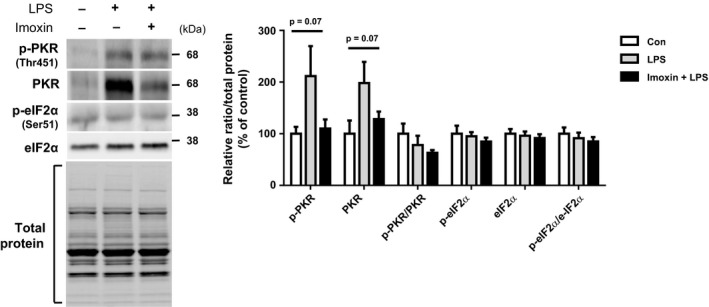
Effects of LPS and imoxin on PKR signaling in gastrocnemius muscle. Gastrocnemius muscle was removed from mice 24 h following LPS injection, with or without pre‐administration of 0.5 mg/kg imoxin (s.c.) 2 h and 24 h prior. PKR signaling was assesses via western blot and representative images and quantification are shown. Values are presented as mean ± SE (n = 6–7/group).

There was no change in TNF‐*α* protein across treatments (ANOVA *P* = 0.3). At the protein level, LPS significantly increased NLRP3 (6.4‐fold) compared to the saline control. Although elevated compared to saline‐treated mice (3.9‐fold), mice receiving imoxin had significantly lower (39%) NRLP3 levels than LPS alone (Fig. 1B). There was no LPS effect on IL‐1*β*; however, both pro‐ and mature IL‐1*β* were significantly lower in Imoxin+LPS then control and LPS (Fig. 1B). There was a tendency for a difference across groups in p‐PKR and total PKR (ANOVA *P* = 0.07 for both), with LPS increasing each by 111% and 99%, respectively (Fig.**** 2). Compared to LPS, p‐PKR and total PKR were reduced by 48% and 33%, respectively, by Imoxin+LPS, although these were not significantly different (Fig.**** 2). There was no effect of treatments on p‐eIF2*α* or total eIF2*α*.

### Muscle atrophy‐related gene and protein expression

**Figure 3:**
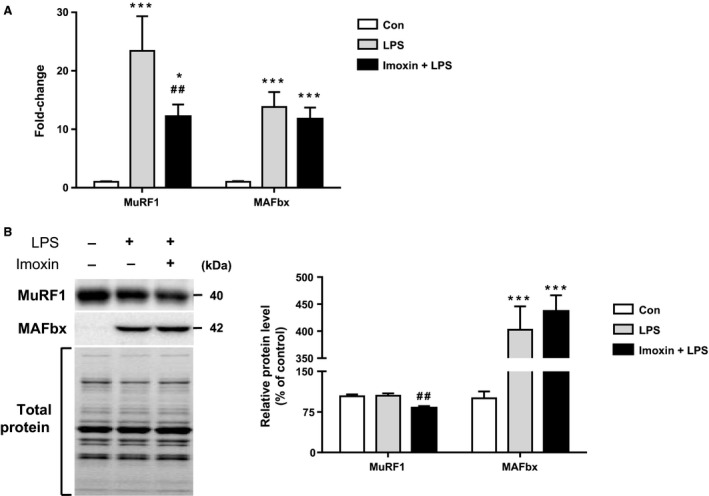
Imoxin attenuates LPS‐induced expression of MuRF1, but not MAFbx in skeletal muscle. Atrogene mRNA (A) and protein (B) expression were quantified from gastrocnemius muscle 24 h following LPS injection or saline (Con). Imoxin was administered (0.5 mg/kg, s.c.) 2 and 24 h prior to LPS (Imoxin+LPS). Data are expressed relative to control mice, and presented as mean ± SE (n = 6–7/group). *P < 0.05 and ***P < 0.001 compared with the Control group, ##*P* < 0.01 compared with the LPS group.

LPS is an established model to induce atrophic signaling through upregulating the transcription of the muscle enriched ubiquitin ligases MAFbx (atrogin‐1) and MuRF1 (Doyle et al. 2011). As expected, LPS increased MuRF1 mRNA compared to control (23‐fold), which was attenuated by >50% in Imoxin+LPS (Fig. 3A). Similarly, LPS increased MAFbx gene expression (14‐fold); however, imoxin did not affect this increase. MuRF1 protein was not increased in response to LPS; however, it was significantly lower, by 18% and 22%, in Imoxin+LPS compared to control and LPS, respectively (Fig. 3B). Protein levels of MAFbx increased ~4‐fold, in both LPS and Imoxin+LPS compared to control.

**Figure 4:**
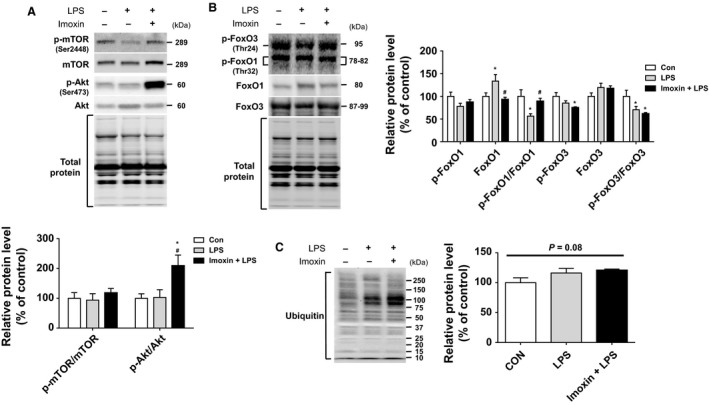
Effects of imoxin on muscle hypertrophy signaling responses. Gastrocnemius muscle was harvested and signaling molecules involved in muscle hypertrophy (A), atrophy (B), and ubiquitinated proteins (C) were assessed via western blot. Data are presented as mean ± SE (n = 6–7/group). *P < 0.05 compared with the Control group, #*P* < 0.05 compared with the LPS group.

**Figure 5:**
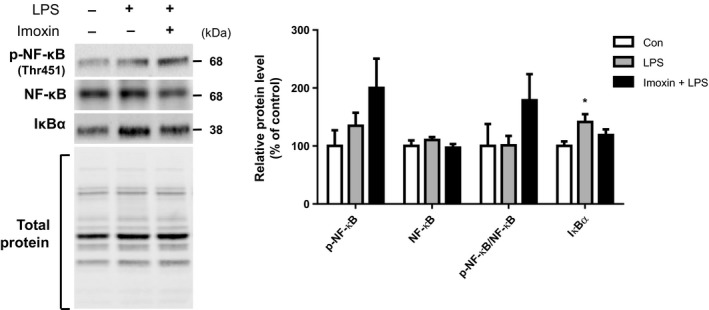
NF‐κB signaling in response to LPS. NF‐κB signaling was assessed via western blot from gastrocnemius muscle harvested 24 h after LPS with or without pretreatment with imoxin. Representative blots are shown. Data are expressed as mean ± SE (n = 6–7/group). *P < 0.05 compared with the Control group.

Neither LPS nor Imoxin+LPS had an effect on phosphorylation of the anabolic molecule mTOR. Although LPS alone had no effect on Akt phosphorylation, Imoxin+LPS had significantly greater p‐Akt compared to both control and LPS mice (Fig. 4A). There were no treatment effects on p‐FoxO1. Total FoxO1 was upregulated by 33% in response to LPS (*P* < 0.05), but this response was completely prevented in Imoxin+LPS. The ratio of p‐FoxO1/FoxO1 was reduced by LPS, and this was prevented in Imoxin+LPS (Fig. 4B). Total FoxO3 did not differ across groups. Phospho‐FoxO3 was lower in Imoxin+LPS compared to control, whereas the p‐FoxO3/FoxO3 ratio was lower in both LPS and Imoxin+LPS, with no difference between the two (Fig. 4B). Despite changes in atrogenes and FoxO signaling, protein ubiquitination only tended to differ across groups (ANOVA *P* = 0.08; Fig. 4C), elevated by 16% and 21% in LPS and Imoxin+LPS, respectively. No treatment effects on phosphorylated or total NF‐*κ*B were observed (Fig.**** 5). I*κ*B protein expression was increased by LPS, but was not elevated in Imoxin+LPS.

## Discussion

The adverse effects of PKR on whole body (Nakamura et al. 2010) and muscle metabolism (Eley et al. 2010) have been described. Many of the deleterious consequences of PKR activity have been attributed to its involvement in inflammatory signaling (Kang and Tang 2012). The PKR inhibitor, imoxin, has anti‐inflammatory effects in the lung, liver, and central nervous system (Tronel et al. 2014; Nakamura et al. 2015; Li et al. 2017), and has benefits on metabolic health (Nakamura et al. 2014). We show here that this finding extends to skeletal muscle, as expression of several inflammatory cytokines induced by LPS, including TNF‐*α*, IL‐1*β*, and NLRP3, were attenuated by pretreatment with imoxin.

Interestingly, PKR appears to regulate NLRP3 inflammasome activity, including maturation and secretion of IL‐1*β* (Lu et al. 2012). However, others have suggested that PKR activity, while essential for some cytokines following LPS stimulation, does not modulate the inflammasome (He et al. 2013; Hett et al. 2013). Elevated NLRP3 has an established role in insulin resistance (Vandanmagsar et al. 2011; Wen et al. 2011) and may compromise muscle function (Boursereau et al. 2018). It was recently shown that NLRP3 knock‐out mice are protected from sepsis‐induced muscle inflammation, atrophy (Huang et al. 2017), and sarcopenia (McBride et al. 2017). To our knowledge, no previous studies have related PKR and NLRP3 in skeletal muscle. Similarly, limited evidence exists regarding the role of imoxin, in vivo, on the skeletal muscle response to an inflammatory insult. We observed a significant increase in muscle NLRP3 protein upon LPS stimulation, which was attenuated in Imoxin+LPS, supporting a potential role of PKR in inflammasome signaling. Similarly, although neither pro nor mature IL‐1*β* was increased by LPS, both were lower in Imoxin+LPS,

Pro‐inflammatory cytokines, including IL‐1*β*, IL‐6, and TNF‐*α*, induce expression the atrogenes MuRF1 and MAFbx, and lead to muscle atrophy (Lang et al. 2002; Frost and Lang 2005; Haddad et al. 2005; Huang et al. 2017). Given our observations that imoxin attenuated inflammation and NLRP3 signaling, we investigated the effect of imoxin on LPS‐induced expression of these genes. As expected, both MAFbx and MuRF1 mRNA were robustly increased by 23‐fold and 14‐fold, respectively, in the group that received LPS alone compared to control (Dehoux et al. 2003). Importantly, pretreatment with imoxin attenuated the increase in MuRF1 compared to LPS alone by approximately ~50%, but had no effect on MAFbx. The observed reduction in MuRF1 could have important implications for blunting muscle atrophy.

Although accelerated muscle protein breakdown plays the dominant role in muscle wasting induced by inflammation, LPS may also impair anabolic signaling and suppress protein synthesis (Vary 1998; Eley and Tisdale 2007; Eley et al. 2008a). Akt has a well‐established role in anabolic signaling, but also opposes protein degradation (Bodine et al. 2001) by suppressing the expression of atrogenes through inhibition of FoxO activity (Stitt et al. 2004). Reductions in phosphorylation status of FoxO (e.g., FoxO1 and FoxO3) by LPS induces nuclear translocation and transcriptional activity, resulting in elevations in MAFbx and MuRF1 (Bodine and Baehr 2014). Inactivation of FoxO can induce hypertrophy and attenuate MuRF1 and MAFbx under septic conditions (Reed et al. 2012). In the current study, Akt phosphorylation, an indicator of Akt activity, was elevated in Imoxin+LPS mice compared to LPS alone. While we did not observe a change in p‐FoxO1, the ratio of p/total FoxO1 was reduced by LPS, but unchanged in Imoxin+LPS. This could suggest a potential reduction in LPS‐induced FoxO1 nuclear translocation and transcriptional activity by imoxin, but cannot be determined in the current study. This finding could potentially explain the blunted MuRF1 observed with Imoxin+LPS, but does not explain the discordant findings between MuRF1 and MAFbx.

The transcription factor NF‐*κ*B is induced by inflammation, and results in elevated transcription of MuRF1 and a muscle wasting phenotype, without affecting MAFbx (Cai et al. 2004). As such, we assessed whether differences in NF‐*κ*B could have accounted for the blunted MuRF1 expression in Imoxin+LPS that was not observed for MAFbx, but observed no differences in NF‐*κ*B across treatments. Typically, under inflammatory stimuli, I*κ*B*α* is degraded, allowing NF‐*κ*B to translocate to the nucleus and induce transcription. With prolonged stimulation, such as the 24 h timepoint assessed here, NF‐*κ*B also induces transcription of I*κ*B, providing negative feedback to suppress NF‐*κ*B (Scott et al. 1993; Hoffmann et al. 2002). Here, we observed an elevation in I*κ*B*α* in LPS that was absent in Imoxin+LPS. The role of NF‐*κ*B on the anti‐catabolic effects of imoxin remains unclear.

Our data are in line with previous reports demonstrating that compounds that induce atrophy, including LPS, consistently increase p‐PKR and reduce protein synthesis in vitro. Use of a catalytically inactive PKR variant and pharmacological inhibition of PKR with imoxin prevent protein degradation and restore protein synthesis disrupted by these various atrophic or inflammatory stimuli (Eley and Tisdale 2007; Eley et al. 2008a,b; Russell et al. 2009; Supinski and Callahan 2011), whereas imoxin alone does not affect protein synthesis (Eley and Tisdale 2007). Similarly, PKR inhibition in vivo has been shown to protect against diaphragm dysfunction (Supinski and Callahan 2011) and cancer cachexia (Eley et al. 2007).

We observed elevated expression of TNF‐*α* and IL‐1*β* whereas others have shown this to be resolved by 24 h (Huey et al. 2008a,b). Our data corroborate that IL‐6 mRNA changes more transiently, returning to baseline levels within 24 h (Huey et al. 2008a), although imoxin‐treated mice had lower IL‐6 than LPS alone. Despite observed elevations, cytokine expression typically peaks early, within 4 h, after LPS exposure (Lang et al. 2003), and precedes increases in MuRF1 and MAFbx mRNA (Murton et al. 2009). Similarly, we did not observe a significant increase in p‐PKR or p‐eIF2*α* 24 h after LPS. Others have reported an earlier peak in p‐PKR following LPS administration, occurring between 4 and 8 h, then returning to baseline (Supinski and Callahan 2011; Li et al. 2017). Our study is limited to this single 24 h timepoint. The reported timecourse of PKR phosphorylation suggests that activity of PKR is transient following a single inflammatory insult, and that PKR activation precedes other inflammatory changes, which remained elevated at 24 h. However, the possibility exists that imoxin may be working through off‐target mechanisms. For example, imoxin has been shown to inhibit CDK activity in neurons, eliciting therapeutic effects independent of PKR signaling (Chen et al. 2008). Imoxin may also exert anti‐inflammatory effects by blunting TLR4 signaling. In the absence of TLR4, macrophages stimulated with LPS had >50% reduction in PKR, and an absence of LPS‐induced p‐PKR (Li et al. 2015).

In the current study, we did not assess contractile function, as this was not expected to decline with this dose of LPS (Meador and Huey 2009). In addition, we have not determined whether skeletal muscle *per se*, or resident immune cells are targeted by imoxin, leading to the observed decrease in LPS‐induced inflammation. Furthermore, our single inflammatory insult did not cause overt loss of muscle or muscle atrophy or significantly elevate protein ubiquitination. Regardless, there appears to be less skeletal muscle inflammation and MuRF1 mRNA in mice receiving imoxin prior to LPS. This finding suggests that imoxin administration may protect skeletal muscle and possibly preserve muscle function, under inflammatory conditions; however, the efficacy of imoxin as a treatment following an inflammatory insult warrants further investigation. Longer‐term studies should be conducted to determine the therapeutic potential of chronic PKR inhibition with imoxin on the preservation of skeletal muscle health. Notably, prolonged daily administration of imoxin, at the dose used in this study, improved metabolic health in a mouse model of diabetes, with no observed ill effects (Nakamura et al. 2014).

## Conclusion

Results of the current study provide evidence that short‐term administration of the PKR inhibitor imoxin attenuates skeletal muscle inflammation and NLRP3 in vivo, in response to a subsequent acute inflammatory insult with LPS. Furthermore, imoxin blunted the LPS‐induced reduction in FoxO1 phosphorylation and increase in mRNA of the atrogene MuRF1. These findings highlight the potential of imoxin to protect against skeletal muscle inflammation and preservation of muscle mass and function, which require further study.

## Conflict of Interest

The authors have no conflicts of interest to report.
